# Differential expression of myometrial AP‐1 proteins during gestation and labour

**DOI:** 10.1111/jcmm.13335

**Published:** 2017-09-25

**Authors:** Lubna Nadeem, Tali Farine, Anna Dorogin, Elzbieta Matysiak‐Zablocki, Oksana Shynlova, Stephen Lye

**Affiliations:** ^1^ Lunenfeld Tanenbaum Research Institute Mount Sinai Hospital Toronto Ontario Canada; ^2^ Department of Physiology University of Toronto Toronto Ontario Canada; ^3^ Department of Obstetrics & Gynaecology University of Toronto Toronto Ontario Canada

**Keywords:** myometrium, pregnancy, preterm labour, rodents, transcription factors

## Abstract

Preterm labour (PTL) is a leading cause of perinatal mortality and postnatal morbidity. Contractions of the uterine muscle (myometrium) that determine the onset of labour depend on the expression of contraction‐associated proteins (CAPs, *i.e*. connexin43) regulated by dimeric AP‐1 transcription factors. Here, we examined subcellular (by immunoblotting) and tissue expression (by immunohistochemistry) of myometrial AP‐1 proteins (cJUN, JUNB, JUND, cFOS, FOSB, FRA1, FRA2) throughout gestation and TL in different species (mouse, rat and human). To identify the critical AP‐1 members associated with preterm birth, we studied their expression in mouse model of ‘infectious’ (LPS‐induced) and ‘sterile’ (RU486‐induced) PTL. We found that (1) myometrial AP‐1 composition is preserved *in vivo* between different species (rodents and human) indicating that Fos/Jun heterodimer (*i.e*. FRA2/JUND) may be indispensable for labour initiation. (2) Our *in vivo* study using murine models of gestation shows that there is a similarity in the myometrial AP‐1 protein composition during TL and pathological PTL of different aetiology suggesting the involvement of similar molecular machinery in the induction of labour. (3) This study is first comprehensive protein analysis of seven AP‐1 members in human labouring *versus* non‐labouring myometrium, showing their cellular expression and tissue distribution in relation to labour status.

## Introduction

Preterm birth (PTB, birth at <37 weeks of gestation) is the most significant problem in clinical obstetrics. It occurs in 5–10% of all pregnancies worldwide and is a major cause of perinatal mortality, morbidity and long‐term disability 1. Preterm infants are 40 times more likely to die compared to the term infants and are threatened with developmental anomalies like cerebral palsy, blindness, deafness and respiratory illness 2, 3, 4, 5. To prevent PTB, it is critical to understand the molecular mechanism of labour initiation. It is now widely recognized that progesterone (P4) is the key regulatory hormone required for the maintenance of pregnancy in all species 6. It maintains the uterine muscle (myometrium) in quiescent state throughout gestation by suppressing the transcription of genes encoding contraction‐associated proteins (CAPs, *i.e*. connexin43 (*GJA1*), prostaglandin receptor (*F2α*), oxytocin receptor (*OXTR*) 7) and inflammatory genes (*i.e. NF‐*κ*B2*,* MCP‐1*,* IL‐1* 8, 9). Recently, we have shown that the molecular mechanism by which P4 suppresses CAPs during pregnancy is through the trans‐repression of the activator protein‐1 (AP‐1) family of transcription factors (TFs) by the progesterone receptors (PRs: PRA and PRB), which physically interact with these factors as well as with transcriptional corepressors p54^nrb^/mSin3A/HDAC that are recruited to the AP‐1 consensus sites 10, 11, 12.

The AP‐1 family consists of four subfamilies; (i) Jun (c‐Jun, JunB, JunD), (ii) Fos (c‐Fos, FosB, Fra1, Fra2), (iii) Mafs (c‐Maf, MafB, MafA, MafG, MafF, MafK, Nrf1 and Nrf2 and (iv) ATF (ATF2, ATF3/LRF1, B‐ATF, JDP1, JDP2). These proteins share structural similarities as they all contain basic leucine zipper (bZIP) region, which facilitates their homo‐ or heterodimerization. AP‐1 dimers recognize either 12‐*O*‐tetradecanoylphorbol‐13‐acetate (TPA) response elements (5′‐TGAG/CTCA‐3′) or cAMP response elements (CRE, 5′‐TGACGTCA‐3′) in the promoter/enhancer region of numerous genes including CAPs 13, 14, 15. Jun and Fos subfamilies are the most potent transcriptional regulators of critical cellular functions. Jun proteins form homo‐ or heterodimers while Fos proteins form Fos/Jun heterodimers 16, which are electrostatically more stable compared to Jun/Jun homodimers and therefore are more potent transcriptional activators 17, 18. We and others reported earlier that AP‐1 TFs regulate myometrial expression of CAPs 19, 20, 21, in particular connexin43 (Cx43) 22, 23, 24; that Fos/Jun heterodimers are stronger inducers of *Cx43* transcription compared to Jun/Jun homodimers; and that their transcriptional activation potential depends upon the specific composition of Fos/Jun heterodimers 10, 25, 26. We have also discovered that (1) Jun and Fos members differentially interact with PRs such that Jun proteins interact with both PRs while Fos proteins show higher interaction with PRA, and (2) in the presence of Jun/Jun homodimers P4‐liganded PRs suppress while in the presence of Fos/Jun heterodimers (*i.e*. FRA2/JUND) unliganded PRA activates Cx43 transcription 10. We suggest therefore that AP‐1 transcription factors orchestrate myometrial function during pregnancy and term labour (TL) by acting as master regulators of genes involved in labour induction in multiple species.

We and others have previously reported that there are significant changes in the myometrial AP‐1 mRNA levels throughout gestation, TL and PTL in rat 23 and mice 27. A comprehensive analysis of their protein expression and tissue localization in the pregnant and labouring myometrium however is lacking. There is no published information regarding the dynamic cellular change in AP‐1 proteins in the course of pregnancy and TL. We suggested that the spatial and temporal distribution of AP‐1 TFs in pregnant and labouring uterus may serve as an indicator of their dimerization status, which is critical to the transcription of CAPs and the onset of labour. The majority of research has been conducted using pregnant rodents due to understandable ethical restrictions in analysis of human pregnant myometrium, which is limited to term pregnant and labouring samples only. The main objective of this study therefore is to examine the expression and cellular/tissue localization of AP‐1 proteins in mouse and rat myometrium throughout gestation, TL and PTL of different aetiology, and to compare it to the AP‐1 expression in pregnant and labouring human myometrium.

## Materials and methods

### Rat model of gestation and labour 23

The Animal Care Committee of the MaRS Animal Research Facility (Toronto, ON, Canada) approved these animal studies (AUP #2379). Virgin female Wistar rats (12–15 weeks, 225–250 g weight) and male Wistar rats (250–300 g weight) were purchased from Charles River Laboratories (St. Constance, QC, Canada) and housed in the MaRS Toronto Medical Discovery Tower Animal Research Facility. Rats were maintained on standard rat chow and water in a 12‐hr:12‐hr light–dark cycle. Male (one male per cage) and female rats (one per cage) were housed separately, and monogamous pairs were only mated overnight. The following morning male and female rats were separated and the day when a vaginal plug was detected was considered day 1 of gestation. Animals were killed by carbon dioxide inhalation and uterine samples were collected in the morning of gestational day (GD) 8, 10, 12, 15, 17, 19, 21, 22, 23 (TL) (*n* = 4). Labour samples (GD23) were collected during active TL following the delivery of at least one pup.

### Mouse model of gestation and labour 28

All mouse experiments were approved by the TCP Animal Care Committee (AUP # 17‐0164H). Hsd:ICR (CD‐1) outbred mice used for these experiments were purchased from Harlan Laboratories (http://www.harlan.com/). All mice were housed under specific pathogen‐free conditions at the Toronto Centre for Phenogenomics (TCP) on a 12L:12D cycle and were administered food and water *ad libitum*. Virgin female mice were mated overnight with males, and the day of vaginal plug detection was designated gestational day (GD) 1 of pregnancy. The average time of delivery was the early morning of GD19. Our criteria for labour were based on delivery of at least one pup from average number of 14 in two uterine horns.

### Experimental design

#### Normal pregnancy and term labour

Mice were killed by carbon dioxide inhalation, and uterine samples were collected in the morning of GD 11, 13, 15, 17, 18, 19 (term not in labour, TNIL) and during active TL. Tissue was collected at 10 a.m. on all days with the exceptions of the labour sample (TL) that was collected once the animals had delivered at least one pup 28. The part of uterine horn close to cervix from where foetus was already expelled was removed and discarded; the remainder was collected for analysis. For each day of gestation, tissue was collected from 3 to 4 different animals.

#### LPS‐induced preterm labour

The lipopolysaccharide (LPS) used for this study was isolated from *Escherichia coli*, serotype 055:B5 (Sigma‐Aldrich, St. Louis, MO, USA). On GD15, mice underwent mini‐laparotomy under general anaesthesia (isoflurane) with intrauterine infusion of 125 μg LPS in 100 μl of sterile saline between two lower amniotic sacs (LPS group) or intrauterine infusion of 100 μl sterile saline (Sham group). Animals (*n* = 3 per group) were killed during LPS‐induced PTL (12–24 hrs after the infusion) or 24 hrs after sham surgery.

#### RU486‐induced preterm labour

On GD15 of gestation, two groups of mice were injected subcutaneously with either RU486 (150 μg in 100 μl corn oil containing 10% EtOH, 17ß‐hydroxy‐11β‐[4‐dimethylaminophenyl]‐17‐[1‐propynyl]‐estra‐4,10‐dien‐3‐ne; mifepristone; Biomol International, Plymouth, PA, USA) or vehicle. PTL was induced in 24 ± 2 hrs. Myometrial samples were collected from RU486‐treated animals after delivery of at least one pup (RU486 group), or 24 hrs after injection of the corn oil/ethanol solvent in control mice (vehicle group; *n* = 4/group) as described earlier 28.

### Animal tissue collection and preparation

Animals were killed at specific gestational days and during TL or PTL; placentae and pups, fat and connective tissue were carefully removed from the uteri. For immunohistochemistry analysis (IHC), half of the uterine tissue (whole uteri) was fixed in 10% neutral‐buffered formalin (NBF, VWR International, Mississauga, ON, Canada) or 4% paraformaldehyde (PFA, Electron Microscopy Sciences, Hartfield, PA, USA) for 24 hrs, dehydrated in ascending concentration of ethanol, cleared in xylene and then embedded in paraffin wax. For biochemical analysis, uterine horns were bisected longitudinally in ice‐cold PBS. The decidua basalis was removed by cutting and decidua parietalis was removed by scraping on ice as described previously 24. Myometrial samples were snap‐frozen in liquid nitrogen and stored at −80°C for protein analysis.

### Collection of human term non‐labouring and labouring myometrial tissues

Healthy pregnant women with a singleton pregnancy undergoing elective caesarean delivery at term (gestational age ≥37 weeks) were recruited as ‘term not in labour’ (TNIL, *n* = 6). Caesarean delivery of ‘term in labour’ (TL, *n* = 6) women was performed after the onset of labour due to a foetal distress syndrome (with regular uterine contractions at 10‐min. interval and cervical dilatation >3 cm). A written consent to participate in the study was obtained from each patient. Myometrial biopsy sample of approximately 1 cm^3^ was excised from the upper margin of the lower uterine segment post‐delivery and washed in ice‐cold PBS. For protein analysis, a small part of the biopsy was snap‐frozen in liquid nitrogen and stored at −80°C; the rest was immediately transferred to 10% neutral‐buffered formalin (Harleco, Baltimore, MD, USA) or 4% paraformaldehyde (PFA, Electron Microscopy Sciences, Hartfield, PA, USA), fixed for 24 hrs at 4°C dehydrated in ascending concentration of ethanol, cleared in xylene and then embedded in paraffin wax for subsequent IHC.

### Protein extraction and immunoblotting

Cytoplasmic and nuclear proteins were extracted from tissues using NE‐PER, Nuclear and Cytoplasmic Extraction kit (Pierce, USA) with freshly added protease and phosphatase inhibitor cocktail (Thermo Fisher Scientific Inc., Waltham, MA USA) as described previously 10. Protein concentration was determined through Pierce™ BCA protein assay kit (Thermo Fisher). Equal amounts of protein (50–60 μg) was run through SDS‐PAGE and transferred to a polyvinylidene difluoride membrane (Trans‐blot Turbo Midi PVDF, Bio‐Rad, USA) using Turbo Trans‐Blot system (Bio‐Rad). Blocking was performed with 5% milk in TBS‐T or 1–5% BSA in TBS‐T depending upon the antibody used and then incubated with primary antibody overnight at 4°C. Primary antibodies used for immunoblotting are listed in Table 1. The membranes were washed with TBS‐T (thrice for 10 min. each) and subsequently probed with horseradish peroxidase‐conjugated secondary antibody at room temperature for 1 hr. Secondary antibodies for rabbit, mouse and goat were purchased from Amersham (dilution range; 1:5000–10,000) and normal IgGs from Santa Cruz, USA. After washing, signals were detected using Luminata HRP‐substrate (Millipore, Etobicoke, ON. Canada) and imaging was performed with VersaDoc imaging system; densitometric analysis of immunoblots was performed using Image Lab software (Bio‐Rad, USA). Membranes were stripped and reprobed with housekeeping proteins to confirm equal loading. Different housekeeping proteins were used to validate fractionation of cytoplasmic and nuclear proteins from mouse, rat and human myometrial tissues based on their expression level and ease of detection. For fractionation control, we used tubulin for mouse/human and PCNA for rat tissues as cytoplasmic proteins marker, while we used Histone H1 for mouse and RAN for rat/human tissues as nuclear protein marker. The relative protein expression post‐densitometry was determined with ERK2 normalization for all species as it was the most stable protein expressed in both cellular fractions (cytoplasmic and nuclear) in all three species.

**Table 1:** Sources and dilutions of antibodies used in this study

Antibody	Catalog #	Company	Size kD	Western	IHC	Diluent	Host
				Dilution	Dilution		
Cx‐43	AB1728	Millipore	20,43	1–1000		1% BSA	Rabbit
c‐JUN	sc‐45	Santa Cruz	39	1–500	1–100	5% milk	Rabbit
JUN B	sc‐73	Santa Cruz	39	1–500	1–100	5% milk	Rabbit
JUN D	sc‐74	Santa Cruz	35,40	1–500	1–100	5% milk	Rabbit
c‐FOS	sc‐52	Santa Cruz	62	1–200	1–50	5% milk	Goat
FOS B	sc‐7203	Santa Cruz	45	1–500	1–100	5% milk	Rabbit
FRA 1	sc‐605	Santa Cruz	40	1–500	1–100	1% BSA	Rabbit
FRA 2	sc‐171	Santa Cruz	48	1–500	1–100	5% milk	Rabbit
α Tubulin	sc‐5548	Santa Cruz	55	1–1000	–	5% milk	Rabbit
RAN	sc‐20802	Santa Cruz	28	1–200	–	5% milk	Rabbit
Histone H1	sc‐67324	Santa Cruz	32	1–200	–	5% milk	Rabbit
PCNA	MCA1558F	Bio‐Rad	34	1–1000	–	5% milk	Mouse
β Actin	3662‐100	BioVision	43	1–1000	–	5% milk	Goat
ERK2	sc‐154	Santa Cruz	42	1–1000	–	5% milk	Rabbit

### Immunohistochemistry

All tissues were sliced at 5 μm thickness and baked overnight. The sections were dewaxed in three changes in xylene for 10, 5 and 5 min., respectively, and then rehydrated in ethanol (100% thrice for 2 min. each, 95% once for 2 min.). The endogenous hydrogen peroxide activity was quenched in 3% H_2_O_2_ solution in methanol for 30 min., and tissue rehydration was continued in the descending grades of ethanol (90%, 80%, 70%, 50% for 30 min. each). Slides were washed with 1xPBS and subjected to antigen retrieval (heating in 10 mmol/l sodium citrate in the microwave), then cooled, washed twice with 1× PBS and blocked at room temperature with Dako blocking serum (Dako Diagnostics Canada Inc., Mississauga, ON, Canada) for 1 hr. Primary antibody incubation was performed overnight at 4°C. Slides were washed thrice in 1xPBS, and biotinylated secondary antibodies (Dako) were used against the species of primary antibody for 1 hr at room temperature. After washing in PBS, streptavidin‐labelled horseradish peroxidase‐conjugated antibody (Dako) was used for 30 min. at room temperature. Next, slides were developed using Dako DAB staining kit (0.02% H_2_O_2_, 0.075% 3,3‐diaminobenzidine solution in 1× PBS). Counterstaining was performed using Harris haematoxylin (Sigma‐Aldrich Corp, USA). Sections were subjected to quick dehydration in ascending grades of alcohol, cleared in xylene and mounted with cytoseal mounting medium. Photographs were taken with Nikon Coolpix 4500 camera attached to a Nikon Light Microscope Eclipse 55*i* (Nikon Canada Inc., Mississauga, ON, Canada). The list of primary antibodies and their concentrations are provided in Table 1. Negative controls were performed by omitting the primary antibodies and using their respective IgGs.

### Statistical analysis

To determine differences between groups, we subjected rat and mouse gestational profiles to a one‐way analysis of variance (anova) followed by Dunnett's multiple comparison test as described earlier 29, 30, 31. Statistical analysis was carried out using Prism software (GraphPad Prism version 4; San Diego, CA) with the level of significance set at *P* < 0.05. Differences between two groups (mouse PTL LPS and RU486 *versus* vehicle and human TNIL *versus* TL) were determined by unpaired *t*‐test.

## Results

### Analysis of AP‐1 proteins in mouse myometrium throughout gestation and term labour

**Figure 1:**
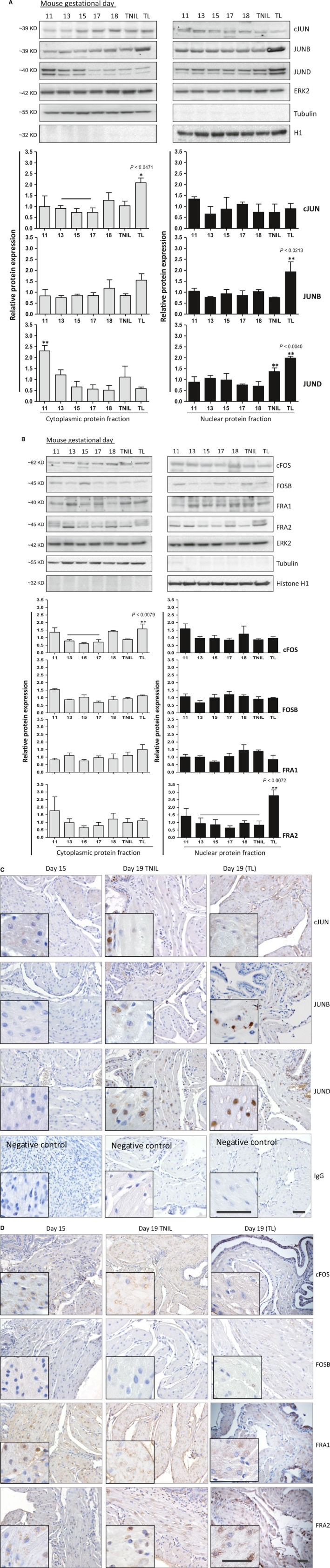
AP‐1 gestational profile in mouse myometrium. Representative Western blots and densitometric analysis of Jun (**A**) and fos proteins (**B**) from mouse myometrium, cytoplasmic (left panel) and nuclear (right panel) fractions. Graphs represent mean of relative protein expression ±S.D., *n* = 4 sets. Cytoplasmic and nuclear proteins were normalized with ERK2 expression. ‘*’ denotes statistical significance of TL *versus* marked gestational days (GD), where TL is compared with all other groups through *Dunnett's multiple comparison test* after *one‐way* anova (*P* values shown on graph). Representative pictures of immunohistochemistry of Jun (**C**) and fos proteins (**D**) from GD15 (mid‐gestation), 19TNIL (term not in labour) and 19TL (term labour) of mouse myometrium. Areas of high magnification are shown as embedded pictures. IgG controls for all GDs are shown in **C**. Scale bar = 50 μm.

Fractionation of mouse myometrium followed by immunoblot analysis of cytoplasmic and nuclear fractions showed that cJUN and cFOS proteins were significantly increased in the cytoplasm during TL (*P* = 0.0471 and *P* = 0.0079, respectively), whereas JUNB, JUND and FRA2 proteins were significantly up‐regulated in the nuclei compared to mid‐gestation (*P* = 0.0213, *P* = 0.0040 and *P* = 0.0072, respectively; Fig. 1A and B). Specific nuclear (Histone H1) and cytoplasmic (tubulin) ‘housekeeping’ proteins were used as fractionation controls. In full accordance with the protein expression results shown on Figure 1A and B, immunolabelling of gestational and labouring mouse myometrial tissues showed pronounced brown deposition of JUNB, JUND and FRA2 proteins in the nuclei of term non‐labouring (GD19, TNIL) and labouring mice (Fig. 1C and D). This immunostaining visually confirmed labour‐related spatial and temporal changes in the intracellular localization of these AP‐1 TFs. We could not detect any changes in tissue localization of cJUN, cFOS, FOSB and FRA1.

### Analysis of AP‐1 proteins in rat myometrium throughout gestation and term labour

**Figure 2:**
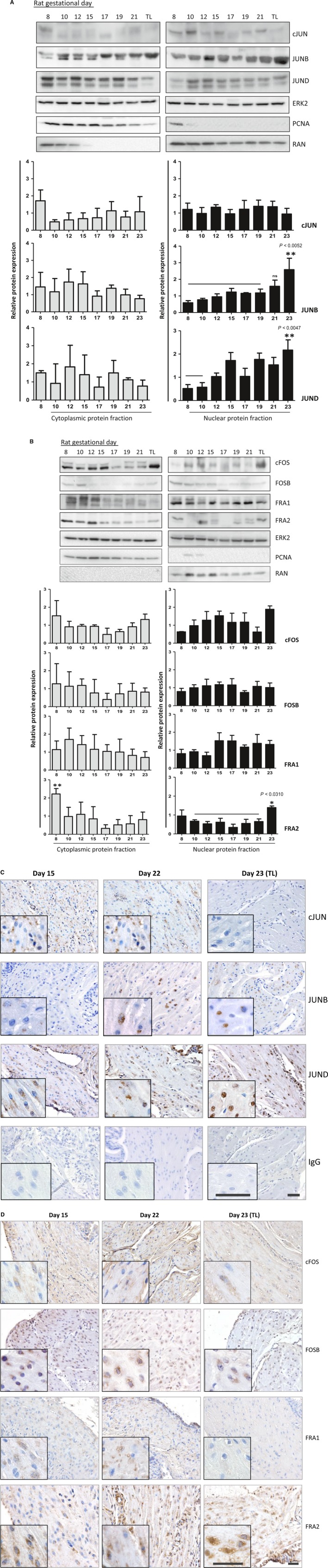
AP‐1 gestational profile in rat myometrium. Representative Western blots and densitometric analysis of Jun (**A**) and fos proteins (**B**) from rat myometrium, cytoplasmic (left panel) and nuclear (right panel) fractions. Graphs represent mean of relative protein expression ±S.D., *n* = 4 sets. Cytoplasmic and nuclear proteins are normalized with ERK2 expression. ‘*’ and ‘**’ denotes statistical significance of TL *versus* marked gestational days (GD), where TL is compared with all other groups through *Dunnett's multiple comparison test* after *one‐way* anova (*P* values shown on graph). Representative pictures of immunohistochemistry of Jun (**C**) and fos proteins (**D**) from GD15 (early gestation), GD22 (TNIL, term not in labour) and GD23 (TL, term labour) of mouse myometrium. Areas of high magnification are shown as embedded pictures. IgG controls for all GDs are shown in **C**. Scale bar = 50 μm.

Similar to mouse myometrium, in rat myometrium significant increase in the expression of nuclear JUNB, JUND and FRA2 proteins was detected during TL as compared to early/mid‐gestation (*P* = 0.0052, *P* = 0.0047 and *P* = 0.0310, respectively). There was a decrease in the cytoplasmic FRA2 (*P* < 0.05) protein at late gestation and during TL as compared to early gestation, while there was no change in the cytoplasmic protein expression of cFOS, FOSB and all JUNs during labour (Fig. 2A and B). Immunohistochemical staining confirmed an increase in nuclear JUNB, JUND and FRA2 protein expression in the labouring rat myometrial tissue (Fig. 2C and D). We noticed a significant increase in brown deposition in nuclei of labouring (GD23) rat myometrium as compared to TNIL (GD22) or mid‐gestation (GD15), indicating an obvious change in tissue localization of JUNB, JUND and FRA2 proteins directly related to term labour contractions.

### AP‐1 expression in term human myometrium: effect of labour

**Figure 3:**
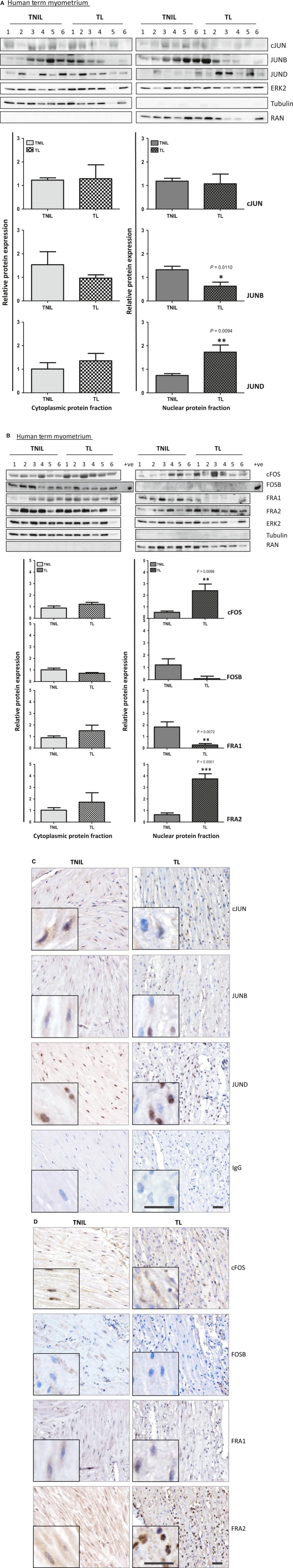
Expression of AP‐1 proteins in human term non‐labouring (TNIL) and labouring (TL) myometrium. Representative Western blots and densitometric analysis of Jun (**A**) and fos proteins (**B**) from human myometrium, cytoplasmic (left panel) and nuclear (right panel) fractions. Graphs represent mean of relative protein expression ±S.D., *n* = 6 samples. Cytoplasmic and nuclear proteins are normalized with ERK2 expression. ‘*’, ‘**’, and ‘***’ denotes statistical significance of TL *versus* TNIL, where TNIL *versus* TL groups were compared using *unpaired t‐test* (*P* values shown on graph). Representative pictures of immunohistochemistry of Jun (**C**) and fos proteins (**D**) from TNIL and TL of human myometrium. Areas of high magnification are shown as embedded pictures. IgG controls are shown in **C**. Scale bar = 50 μm.

In the current study, we performed full and detailed analysis of all seven AP‐1 proteins cellular expression and tissue localization. In human term myometrial tissues from non‐labouring and labouring women, we found no significant change in the expression of cytoplasmic Jun and Fos proteins; however, a significant increase was recorded in the expression of nuclear JUND, cFOS and FRA2 (*P* = 0.0094, *P* = 0.0098 and *P* = 0.0372, respectively, Fig. 3A and B). FOSB protein was almost undetectable in the nuclear fraction of term myometrium (the Abs specificity was confirmed by the detection of positive control alone, Fig. 3B), while the level of nuclear FRA1 and JUNB was decreased (*P* = 0.0072 and *P* = 0.0110, respectively). Additionally, we performed immunohistological examination of human myometrial tissues to confirm the intracellular expression and spatial distribution of all seven AP‐1 proteins. Jun proteins (cJUN, JUNB and JUND) were always detected in the nucleus of labouring myometrial SMCs (Fig. 3C). *In situ* localization of Jun proteins in the myometrial tissue revealed that nuclear JUND protein was profoundly increased during human TL as compared to TNIL myometrium while cJUN expression and tissue localization were unchanged by the labour status. Consistent with our protein expression results, intense nuclear cFOS and FRA2 staining was detected in human labouring samples as compared to non‐labouring tissue (Fig. 3C). The immunostaining of both FOSB and FRA1 in human non‐labouring and labouring myometrial samples was weakly detectable (Fig. 3C).

### Myometrial AP‐1 protein expression during preterm labour

**Figure 4:**
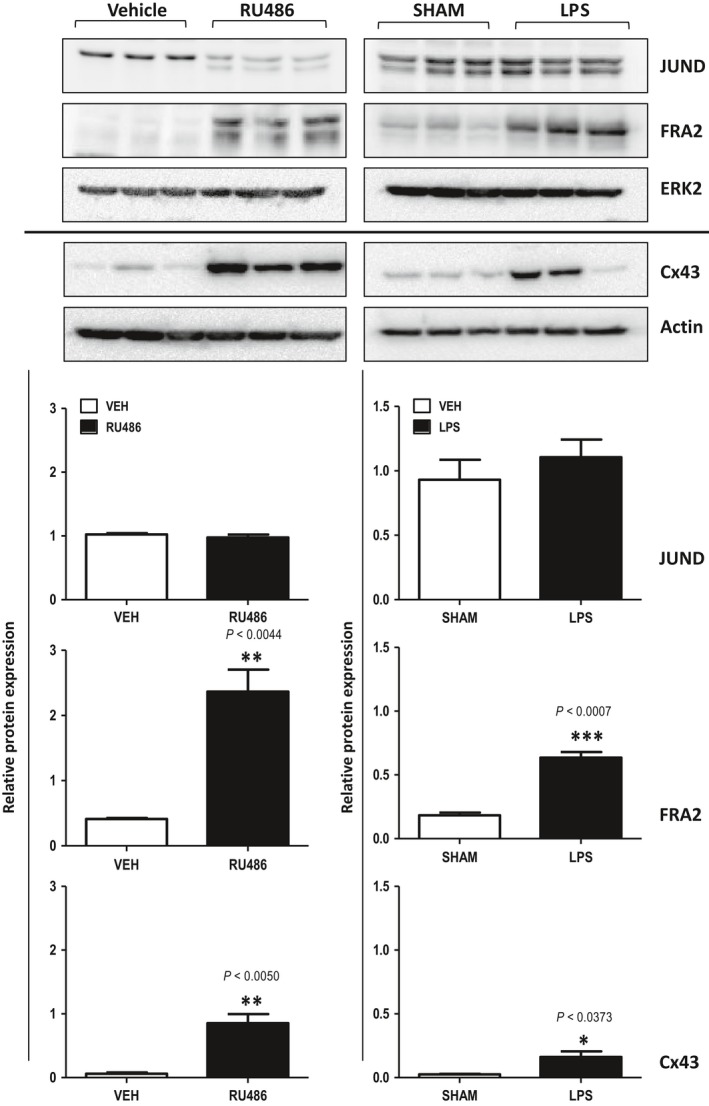
Fra2 protein is up‐regulated in mouse models of preterm labour. Preterm birth was induced in mice using intraperitoneal injection of P4 antagonist RU486 (150 μg) or intrauterine infusion of LPS (125 μg). Nuclear and cytoplasmic proteins were extracted from the myometrium of labouring mice and their age‐matched controls and subjected to SDS‐PAGE and Western blot analysis. Left panel) RU486 model representing GD16 with vehicle (non‐labouring) and RU486 (labouring *n* = 4) Right panel) LPS‐induced preterm model represents GD16 with sham (non‐labouring) and LPS (labouring, *n* = 3). JUND and FRA2 proteins were detected in nuclear fraction and normalized with Histone H1 while Cx43 protein was detected from cytoplasmic fraction, which is normalized with Actin to determine relative expression. Graphs represent densitometric analysis showing mean ± S.D.; ‘*’, ‘**’, and ‘***’ denotes statistical significance (P values shown on graph). *Unpaired t‐test* was used to determine the differences in protein expression among the treatment *versus* control group.

We then examined PTL induced in GD15 pregnant mice (1) by artificial progesterone blockade using RU486/mifepristone (non‐infectious, ‘sterile’ PTL 32) or (2) by intrauterine infusion of 125 μg LPS (the well‐known model of ‘infectious’ LPS‐induced PTL 33). Both treatments resulted in PTL within 24 hrs with no maternal mortality. Expression of two AP‐1 proteins significantly up‐regulated in the nuclear fraction of mouse term labouring myometrium (JUND and FRA2) was analysed by immunoblotting in myometrial samples collected during PTL. In accordance with our hypothesis, we found a similarity between TL and PTL: a significant up‐regulation of FRA2 was induced by blocking P4 signalling with RU486 and by LPS‐induced intrauterine inflammation irrespective of the trigger that initiated PTL (*P* ≤ 0.05); in contrast to TL, myometrial expression of JUND remained unaltered in both mouse models of PTL (Fig. 4A). In addition, we confirmed that similar to TL, RU486 and LPS treatment which caused an increase in nuclear FRA2 protein expression is associated with significant increase in the expression of CAP Cx43 protein (*P* < 0.05, Fig. 4).

## Discussion

We provide here substantial evidence that the composition of AP‐1 proteins in myometrial cells during normal gestation is a dynamic process, which can influence the initiation of labour. Firstly, we have demonstrated *in vivo* that myometrial AP‐1 composition is preserved between different species (rodents and human), pointing to the specific Fos/Jun heterodimer (*i.e*. FRA2/JUND) that may be indispensable for a labour initiation. Secondly, our current *in vivo* studies using murine models of gestation indicate that there is a similarity in the myometrial AP‐1 protein composition during TL and PTL suggesting that similar molecular machinery is involved to prime the myometrium for labour contractions in case of normal TL and pathological PTL of different aetiology. Thirdly, this study is the first comprehensive protein analysis of seven AP‐1 members in human labouring *versus* non‐labouring myometrium, showing their cellular expression and tissue distribution in relation to labour status.

AP‐1 is a key transcriptional regulator of the gap junction protein Cx43 in rodents and humans that contributes to the initiation of labour 22, 24, 25. Based on our previous animal studies and those of others, we suggest that AP‐1 activation is required to drive the transcription of Cx43 to facilitate myocyte coupling and connectivity, which occurs prior to the onset of active myometrial contractions 34. We have previously demonstrated that myometrial expression of Jun and Fos transcripts do not fluctuate during rat gestation, except FRA2, which is significantly up‐regulated two days prior to TL, while all Fos members (cFOS, FOSB, FRA1 and FRA2) as well as cJUN and JUNB mRNA are elevated during active labour 23. In the mouse myometrium, we found stable expression of Fos proteins FRA1 and FOSB throughout gestation, with the up‐regulation of cytoplasmic cFOS and cJUN as well as nuclear FRA2, JUNB and JUND during labour. We speculate therefore that dominance of Fos/Jun heterodimers prevails during labour as Fos members are capable of forming electrostatically stable heterodimers with Jun compared to weak Jun/Jun interaction 35. These Jun/Jun homodimers dominate during early and mid‐gestation when Fos protein levels are stably low. It is possible that the MAPK intracellular signal transduction pathway is involved in up‐regulation of AP‐1 expression and/or activation, as we found earlier that MAPK proteins ERK and JNK are activated at term in rodents’ myometrium 36. Importantly, FRA2 protein expression and activity are regulated by MAPK, in particular MAPK/ERK‐dependent phosphorylation was shown to increase the DNA binding activity 37, 38, and trans‐activating potential of FRA2 39. Our recent results showed that the changing composition of AP‐1 proteins (from Jun/Jun homodimer during most of gestation to Fos/Jun heterodimer during labour) and the resulting change in their affinity to PR isoforms (PRB during gestation *versus* PRA during labour) are able to switch PR function from being a trans‐repressor to a trans‐activator of Cx43 gene expression 10. Therefore, we speculate that MAPK‐induced FRA2 protein expression in combination with simultaneous increase in JUNB and JUND may cause a formation of stable FRA2/JUND or FRA2/JUNB heterodimers before and during labour, which (in combination with increased PRA:PRB ratio 10) are capable of inducing Cx43 gene expression. This sequence of events provides an essential condition for the activation of myometrium and development of coordinated labour contractions. In addition to PRs, our previous published results clearly indicate the presence of other molecular players capable of switching on/off CAP gene transcription in myometrium 11, 12.

Our present study shows that similar molecular pathways operate in human and animal pregnancy. Currently, very limited and scattered information is available regarding labour‐related changes in human myometrial AP‐1 proteins. Previous studies reported an increase in cFOS 27 and cJUN genes 40, as well as selected AP‐1 proteins in term labouring human myometrium 41 as compared to term not labouring samples. The current study clearly shows that cFOS protein is up‐regulated exclusively in the nucleus of human myometrial cells; moreover, in contrast to previous publications 41, we also detected a significant increase in nuclear JUND expression, while nuclear JUNB expression was decreased. We report now that during TL nuclear expression of FRA1 is decreased, whereas FRA2 protein significantly increased. Our protein expression data are supported by comparative immunohistological analysis of all seven members of Fos and Jun families in term non‐labouring and labouring human myometrium. Intensive brown immunostaining of nuclear FRA2 and JUND proteins during human labour suggest that these two proteins coexist as a FRA2/JUND heterodimer and potentially activate Cx43 transcription 10.

In almost all species, a decrease in tissue/plasma P4 levels occurs prior to the onset of TL. Animal studies have shown that experimental removal of the source of P4 during gestation by ovariectomy or antagonism of PR signalling by RU486 results in termination of pregnancy 28, 32. In contrast, when P4 is injected in pregnant rats to prevent its systemic withdrawal, TL in rat is delayed 23. In women, there is no evidence for a pre‐partum fall in plasma or tissue P4, but the administration of RU486 still leads to increased uterine activity suggesting that P4 plays a similar role in the human as in animals 42. Here using a rodent model, we found that PTL (induced by RU486) is associated with significant up‐regulation of FRA2, which likely contributes to the formation of Fos/Jun heterodimers in the nuclei of myometrial cells, initiating Cx43 protein expression, gap junction formation and labour contractions. In control pregnant mice, however, myometrial Jun/Jun homodimers are dominant suggesting their role in the maintenance of gestation.

Labour in human 43, 44, 45 and rodents 28 is associated with a physiological uterine inflammatory response. Our current study using an LPS model of PTL suggests that infection/inflammation may also increase AP‐1 protein expression. Our data are in agreement with the previous report from MacIntyre et al., that showed an increase in phosphorylated cFos and cJun protein expression in the mouse myometrium 1 hr after intrauterine injection of LPS 34. Importantly, they also reported an increased level of phosphorylated JNK/MAPK 34. Our recent *in vivo* 28 and *in vitro* 46 reports indicate that at term mechanical stretch caused by the growing foetus may lead to the activation of inflammatory response pathways. We also reported that mechanical stretch increases mRNA expression of Fos (cFos, FosB and Fra1) and Jun (cJun and JunB) in rat myometrium 23, 47. In agreement with our data, the pro‐inflammatory cytokine TNFα was found to increase AP‐1 transcriptional activity of human myometrial cells 41. Conversely, AP‐1 is capable of stimulating IL‐8 chemokine expression in human myometrium, and inhibition of AP‐1 by siRNA reduced IL‐8 secretion 48. Given that ours and previous data show: (i) an increase in JUND, cFOS and FRA2 proteins during labour, (ii) an increase in multiple pro‐inflammatory cytokines 49 and (iii) activation of AP‐1 by cytokines 50 in labouring human myometrium, it is feasible to speculate that myometrial CAPs are regulated by pro‐inflammatory proteins *via* AP‐1 activation.

In conclusion, we propose that throughout pregnancy Jun proteins are stably expressed in the uterine myometrial cells, both in the cytoplasm and nuclei, where they form Jun/Jun homodimers which take part in suppressing labour gene (*i.e*. Cx43) expression by interacting with P4‐liganded PRB and transcriptional corepressors. At term, a concomitant increase in Fos, Jun and PRA proteins caused by the local (in human) or systemic (in rodents) withdrawal of P4, results in the assembly of a complex comprising PRA and Fos/Jun heterodimer, which activates Cx43 transcription, thus triggering myometrial activation and labour. Moreover, we suggest that this scenario may be valid for all labour‐associated genes regulated by the AP‐1 transcription factors including other CAPs, pro‐inflammatory cytokines and extracellular matrix proteins. We also suggest that AP‐1 TFs could serve as potential therapeutic target in the management of infection‐associated and idiopathic PTL. Further study is needed to explore these potential regulations.

## Conflict of interest

The authors declare no conflict of interest.
